# Invasive Fungal Infection Complicating Treatment With Ibrutinib

**DOI:** 10.7759/cureus.16009

**Published:** 2021-06-29

**Authors:** Pramuditha Rajapakse, Manish Gupta, Rewaida Hall

**Affiliations:** 1 Internal Medicine/Hematology and Oncology, Danbury Hospital, Yale School of Medicine, Danbury, USA; 2 Internal Medicine, Danbury Hospital, Yale School of Medicine, Danbury, USA

**Keywords:** ibrutinib, invasive fungal infections, tyrosine kinase inhibitors, chronic lymphocytic leukemia, cerebral aspergillosis

## Abstract

Ibrutinib is a selective Bruton’s tyrosine kinase inhibitor (BTKi) approved for the treatment of chronic lymphocytic leukemia (CLL) and other B-cell malignancies. Invasive fungal infections (IFIs) have recently been reported in patients on BTKis despite the absence of significant immunocompromise raising great interest among oncologists regarding the mechanism by which BTKi's permit fungal infections. Here, we describe a fatal case of cerebral aspergillosis in a patient with relapsed CLL while on treatment with ibrutinib. There are few hypotheses on the mechanism by which ibrutinib permits fungal infections. As it becomes more widely used in B-cell cancers, clinicians should be aware of the potential for decreased anti-fungal immunity with this drug.

## Introduction

Ibrutinib is a selective Bruton’s tyrosine kinase inhibitor (BTKi) that was initially approved in 2013 by the US Food and Drug Administration (FDA) for treatment of mantle cell lymphoma and subsequently for the treatment of CLL, Waldenstrom macroglobulinemia, marginal zone lymphoma, and chronic graft‐versus‐host disease [1,2]. Although considered to be less immunosuppressive than conventional immunochemotherapy, invasive fungal infections (IFIs) have recently been reported in patients on BTKi's [3,4]. Invasive fungal infections are life‐threatening infections that can affect multiple organ systems. Underlying conditions that compromise the immune responses to inhaled fungal species serve as risk factors for IFIs. Classic risk factors include severe and prolonged neutropenia, high doses of glucocorticoids, and other drugs or conditions that lead to chronically impaired cellular responses (eg, immunosuppressive regimens administered to treat autoimmune diseases and to prevent organ rejection, AIDS) [5]. An increasing number of reports of IFIs and other opportunistic infections during ibrutinib treatment have been reported over the last few years raising a concern if there is an association between ibrutinib and antifungal immunity [1,3].

## Case presentation

We report a case of a 53-year-old male with a past medical history of CLL who presented to the hospital with persistent fever, cough, and generalized weakness for two weeks. He was diagnosed with CLL in 2014 and was initially treated with bendamustine and rituximab. Then in 2015, he was found to have a relapse, which was treated with Imbruvica. He had been on ibrutinib for five years for relapsed CLL. He was an avid gardener and was very functional with daily activities before the onset of symptoms. On arrival at the emergency department, he was febrile with a temperature of 37.9°C. He was tachycardic with a heart rate of 106. Physical exam was negative for focal neurologic findings. Initial laboratory evaluation showed leukocytosis with a white blood cell (WBC) count of 132.8 x 10^9^/L (range 3.5-10 x 10^9^/L), absolute lymphocyte count of 99.47 x 10^9^/L (range 1-4 x 10^9^/L ), absolute neutrophil count of 6.87 x 10^9^/L (range 2-7.5 x 10^9^/L), thrombocytopenia with a platelet count of 84.5 x 10^9^/L (range 150-400 x 10^9^/L). Complete blood count (CBC) one month prior to the presentation was normal. Serum chemistry showed elevated creatinine at 1.41 mg/dL (range 0.67-1.23 mg/dL) and normal liver function tests. Due to the concern of sepsis given the ongoing treatment with ibrutinib, the patient underwent an infectious workup. Blood cultures yielded no growth, chest x-ray, urinalysis, and computed tomography (CT) of the chest abdomen, and pelvis was unremarkable.

**Figure 1:**
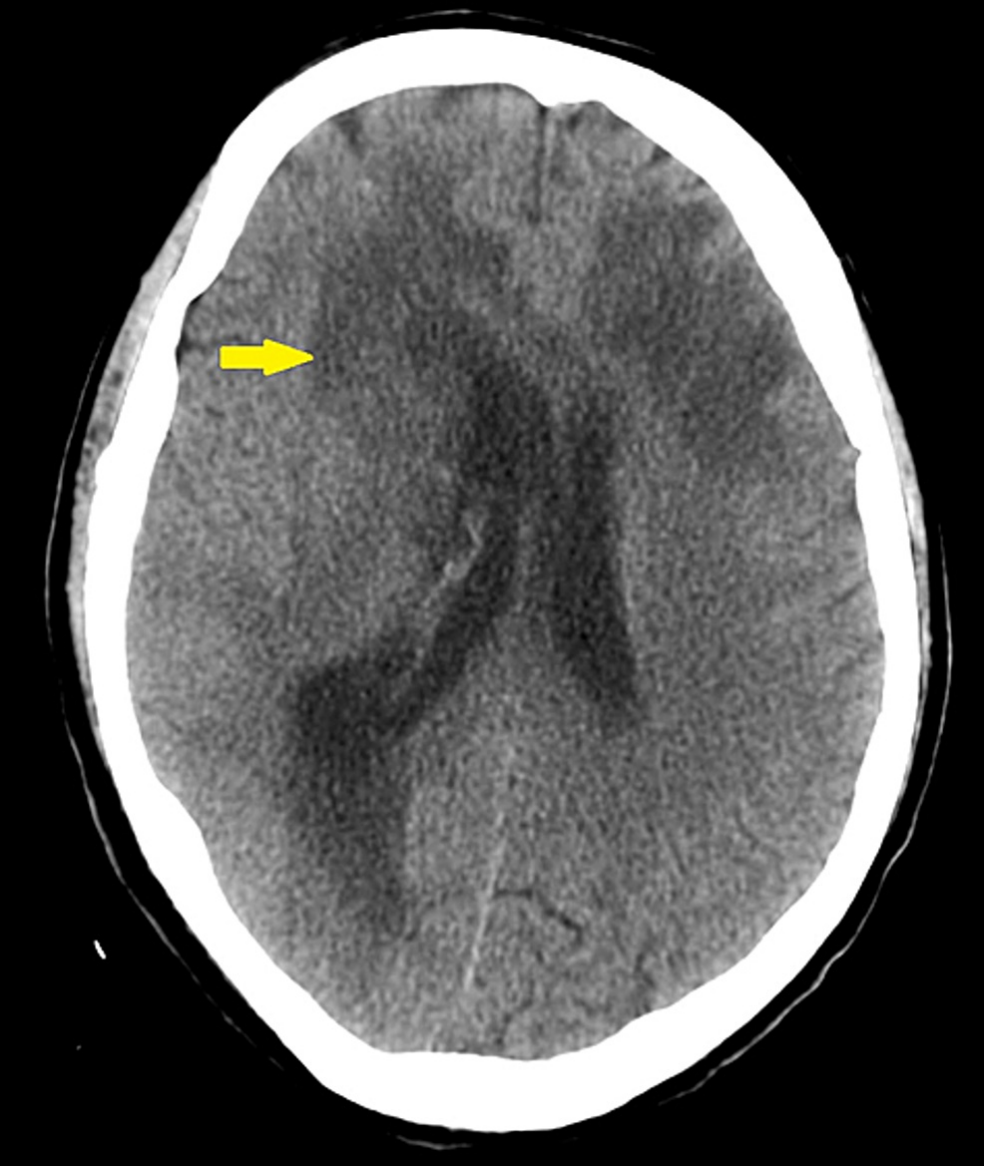
Computed tomography (CT) scan of the head without intravenous contrast with findings concerning the development of a brain abscess particularly on the right where there was mass effect, midline shift, and occlusion of the right lateral ventricle with mild dilatation of the right lateral ventricle.

Two days later, the patient developed acute onset confusion and global aphasia. This prompted further studies including a CT scan of the head without intravenous (IV) contrast. CT scan findings were suggestive of a brain abscess, which included midline shift, and occlusion of the right lateral ventricle with mild dilatation of the right lateral ventricle (Figure 1).

**Figure 2:**
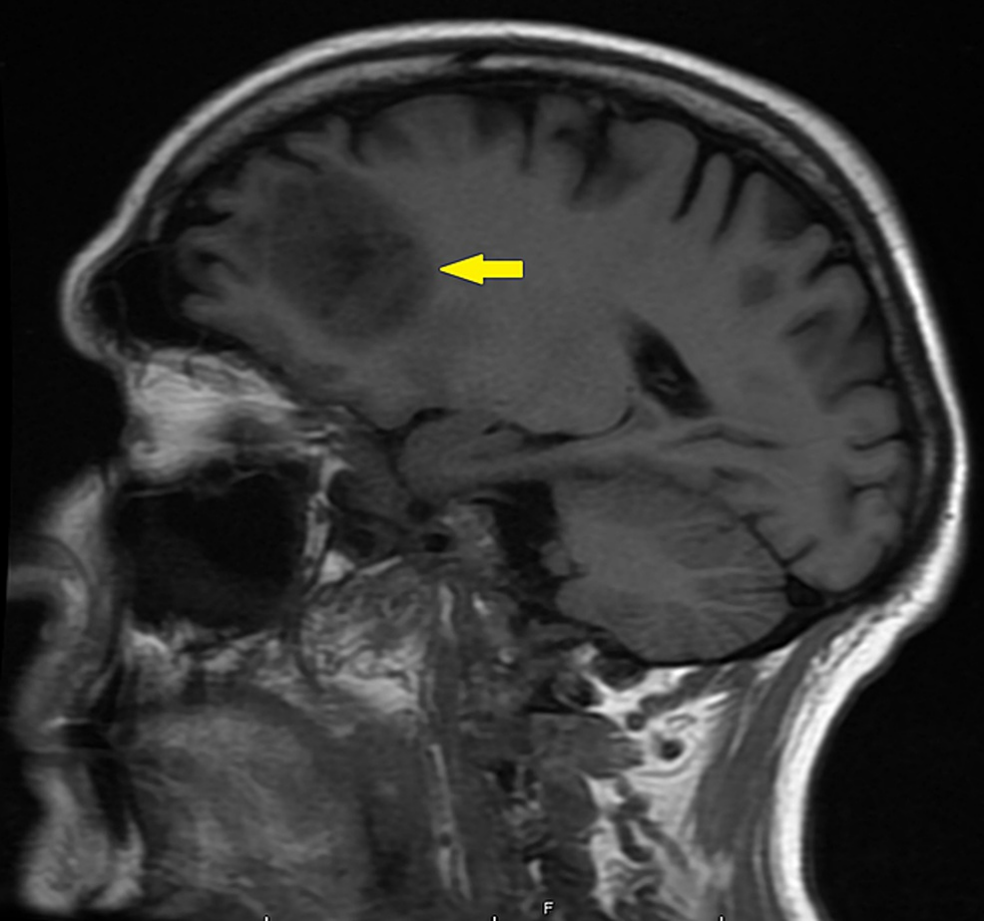
MRI brain with contrast showing mass-like peripherally enhancing lesions in the left frontal lobe.

**Figure 3:**
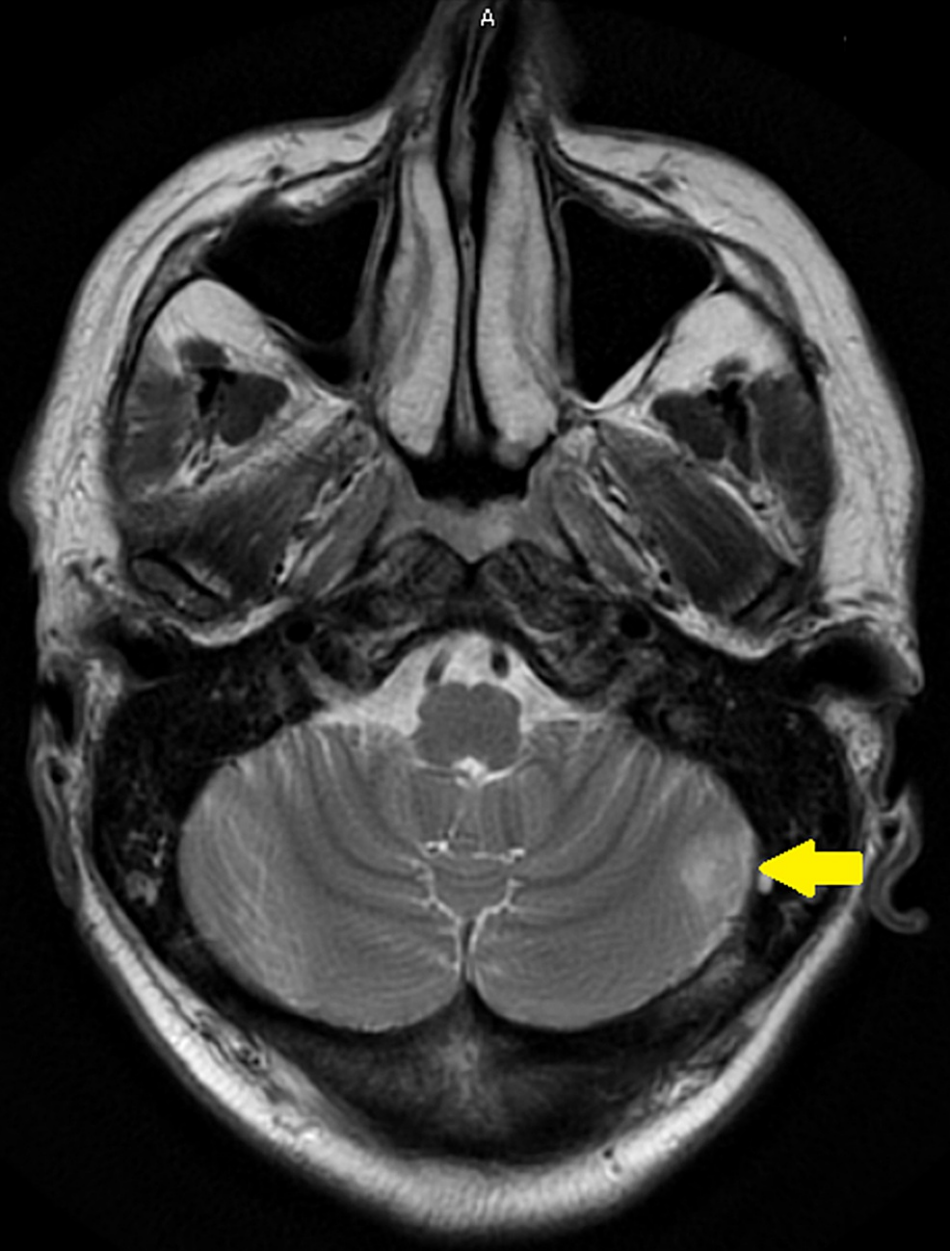
MRI brain with contrast showing a non-enhancing mass-like lesion in the left cerebellum.

Magnetic resonance imaging (MRI) with contrast showed mass-like peripherally enhancing lesions in the left frontal lobe and a non-enhancing mass-like lesion in the left cerebellum, and there was an extensive infiltrative process in the right frontal lobe that involved the corpus callosum, septum pellucidum, likely invading the ependymal surface of the right lateral ventricle (Figures 2 and 3).

The patient was transferred to the intensive care unit for close monitoring. Infectious disease, Hematology/Oncology, and Neurosurgery teams were consulted. The patient was managed initially with broad-spectrum antibiotics including vancomycin, ceftriaxone, and metronidazole. The patient was also started on IV dexamethasone. Levetiracetam was initiated for seizure prophylaxis. Three days after admission, he underwent a left frontal stereotactic craniotomy and resection of the brain. Examination of frozen pathology sections showed fungal hyphae within this necrotic brain tissue highlighted by Grocott-Gomori’s methenamine silver (GMS) stain and was characterized by acute-angle branching and transverse septations. This morphology is consistent with Aspergillus species and therefore a diagnosis of necrotizing aspergillosis was made after histopathological evaluation. The clinical course was further complicated by the development of left hemiparesis and worsening encephalopathy. Subsequently, he was intubated for airway protection. The patient also underwent emergent burr hole and ventriculostomy placement due to imaging findings showing periventricular edema with mass-effect and sub-falcine herniation. He was started on voriconazole and amphotericin B. The patient was successfully extubated a day later. However, encephalopathy continued to worsen. His prognosis remained guarded, and the family made the decision to transition him to comfort measures. The patient expired two weeks later.

## Discussion

This report supports the possible association between treatment with ibrutinib for CLL or non-Hodgkin lymphoma (NHL) and the development of IFIs. Ibrutinib‐associated IFIs have been described in several case reports and a series of patients [1,3]. BTK inhibition can make the patients susceptible to invasive aspergillosis as evidenced by pre-clinical studies [6]. BTK is an important mediator of NF-κB (nuclear factor k beta), which is a major transcription factor that regulates genes responsible for both the innate and adaptive immune response. It plays a critical role in immune surveillance of fungal spores and acts as the primary defense against fungal infections, especially in the lung and central nervous system (CNS). BTKi's cause functional defects in fungal immune surveillance, by inhibition of myeloid cells [7]. BTK signaling also regulates effector functions of myeloid cells, including chemotaxis adhesion, transmigration, reactive oxygen species production, and cytokine response [8]. However, myeloid dysfunction alone does not appear to be sufficient to account for ibrutinib‐associated IFI as only a small percentage of patients treated with BTKi's have developed this. Multiple predisposing factors have been linked to the development of IFI including neutropenia, the use of steroids, diabetes mellitus, and liver disease [9]. CNS aspergillosis generally occurs as a result of direct invasion from adjacent structures or through hematogenous spread. Ibrutinib has good penetration of the blood‐brain barrier, and after entering the CNS, ibrutinib could potentially inhibit CNS macrophages thus eliminating fungal immune surveillance [10].

The largest study that evaluated this association between ibrutinib and IFI is from Israel and included 35 patients. A male predominance, an underlying diagnosis of CLL was seen in most patients, with a notably high percentage of CLL patients with high‐risk features such as 17p deletions or TP53 mutations. But only a few had neutropenia. Aspergillus species was the most common pathogen, with frequent CNS dissemination. Symptoms started shortly after initiating treatment with ibrutinib, in some as early as one day [11]. The number of reports of IFI with ibrutinib appears to be increasing. Early occurrence after starting ibrutinib has been suggested to be related to colonization by fungi prior to starting ibrutinib and may be supported by the short Tmax of ibrutinib (1‐2 hours) [6,8]. A recent publication reported 16 (4.2%) IFIs out of 378 CLL and NHL patients treated with ibrutinib over five years, the majority of whom lacked classic clinical risk factors for IFI. The patients had an unusually high rate of IFI involving the CNS (49%), and multiorgan involvement (60%) predominantly by molds. They had a very high mortality rate of 69% [12].

A literature review of previous case reports reveals that most patients were not neutropenic in the month prior to initiation of treatment with ibrutinib, similar to our patient [12,13]. Notably, the symptoms of IFIs often appeared very early after treatment was initiated in most cases in contrast to our patient who developed IFIs years later. Aspergillus species was the most common pathogen [14]. Despite prompt initiation of appropriate antifungal therapy, mortality was extremely high in previous cases similar to our patient [11]. This emphasizes the importance of early recognition. Staging CT scans are an opportunity to diagnose subclinical fungal infections. Larger studies are needed to attain a better understanding of ibrutinib‐associated IFI and to identify patients at risk, who may benefit from intensified monitoring or chemoprophylaxis. In the interim, there is no recommendation for prophylaxis [15]. When IFI is suspected, it is recommended to consider stopping ibrutinib until the diagnosis is ruled out or the infection has been brought under control.

## Conclusions

Clinicians should be aware of the potential for decreased anti-fungal immunity and the risk for aspergillus infections with ibrutinib, even in the absence of clinically overt immune suppression. The overall incidence of these infections is low enough that routine prophylaxis for all patients with CLL who are taking ibrutinib would be unnecessary. However, prophylaxis is reasonable in select patients with additional risk factors. We would like to make the clinicians aware of this association because early recognition and prompt treatment are critical to prevent life-threatening complications.
